# Effects of Fasting on THP1 Macrophage Metabolism and Inflammatory Profile

**DOI:** 10.3390/ijms25169029

**Published:** 2024-08-20

**Authors:** Julia Rius-Bonet, Salvador Macip, Marta Massip-Salcedo, Daniel Closa

**Affiliations:** 1 Department of Experimental Pathology, Institut d’Investigacions Biomèdiques de Barcelona, Consejo Superior de Investigaciones Científicas (IIBB-CSIC), Institut d’Investigacions Biomèdiques August Pi i Sunyer (IDIBAPS), 08036 Barcelona, Spain; 2 FoodLab, Faculty of Health Sciences, Universitat Oberta de Catalunya, 08018 Barcelona, Spain; 3 Mechanisms of Cancer and Aging Laboratory—South, Josep Carreras Leukaemia Research Institute (IJC), Badalona, 08916 Barcelona, Spain; 4 Mechanisms of Cancer and Aging Laboratory, Department of Molecular and Cell Biology, University of Leicester, Leicester LE1 7RH, UK

**Keywords:** fasting, intermittent fasting, macrophages, inflammation, rheumatic diseases

## Abstract

Fasting can affect the body’s inflammatory response, and this has been linked to potential health benefits, including improvements for people with rheumatic diseases. In this work, we evaluated, in vitro, how changes in nutrient availability alter the inflammatory response of macrophages. Macrophage-differentiated THP1 cells were cultured, deprived of FCS or subjected to cycles of FCS deprivation and restoration to mimic intermittent fasting. Changes in the macrophage phenotype, the cells’ response to inflammatory stimuli and the level of mitochondrial alteration were assessed. The results indicate that while periods of serum starvation are associated with a decrease in IL1β and TNFα expression, consistent with an anti-inflammatory response, intermittent serum starvation cycles promote a pro-inflammatory phenotype. Rapid changes in reducing capacity and mitochondrial response were also observed. Of note, while some changes, such as the production of oxygen free radicals, were reversed with refeeding, others, such as a decrease in reducing capacity, were maintained and even increased. This study shows that different fasting protocols can have diverging effects and highlights that time-limited nutrient changes can significantly affect macrophage functions in cell cultures. These findings help elucidate some of the mechanisms by which specific fasting dietary interventions could help control inflammatory diseases.

## 1. Introduction

Different lifestyle behaviors are being studied in disease management for rheumatic diseases, with a recent specific focus on the therapeutic implications of dieting [1,2,3,4,5]. It has been reported that diets involving changes in the availability of energy, such as calorie-restricted diets or varying fasting protocols, including intermittent fasting, have health benefits for people with rheumatic diseases, partly by targeting the body’s inflammatory state [6,7,8,9,10]. However, the specific mechanisms by which these diets could be altering inflammation are still unclear.

Macrophages are immune cells that are found in every tissue and are involved in a broad range of physiological functions. They are known to be key in inflammation, innate immune host defense, maintenance of tissue homeostasis, the clearance of cellular debris and tissue repair, among many other functions [11]. Due to their heterogeneous role, macrophages have the ability to enter different activation states that allow for a rapid response to changes in the microenvironment. For a long time, the polarization state of macrophages has been simplistically classified as pro-inflammatory (M1) or anti-inflammatory (M2), taking the model of the Th1 and Th2 responses of lymphocytes [12]. It is now clear that this classification masks a huge range of different adaptive responses. Macrophage profiles are dynamic, fluctuating between phenotypes, and depending on their activation state, the expression levels of cytokines, enzymes and other markers vary, which alters macrophage activity [13].

The acquisition of an M1 or M2 phenotype results in significant changes in the metabolic pathways used by macrophages to obtain energy. While macrophage M1 activation is associated with increased glycolysis; an impaired tricarboxylic acid cycle (TCA); and oxidative phosphorylation, M2 activation is primarily dependent on fatty acid oxidation to maintain TCA at full capacity [14]. However, the link between phenotype and metabolism works in both directions, and changes in glycolysis enzyme activity, such as pyruvate kinase or glyceraldehyde 3-phosphate dehydrogenase, have been shown to have effects on the expression of inflammatory cytokines such as IL-1β and TNFα [14,15]. This correlation can be important in situations where there is a limitation or a fluctuation in the availability of nutrients, as occurs in certain diets.

On the other hand, nutrient deprivation is also a stressful situation that induces important changes in the activation level of macrophages, which respond not only to the lack of energy substrate but also to that of growth factors, vitamins or structural components [14]. In vitro studies have shown that removing fetal serum from the culture medium induces macrophage activation, phagocytosis, apoptosis and changes in different signaling pathways despite maintaining glucose availability [16,17]. These changes may be relevant to understanding potential responses to diets that include extended fasting or those based on shorter fasting cycles, such as the intermittent fasting (IF) diet. Indeed, the response of macrophages to short-term starvation and repetitive cycles of starvation is an important issue, not only in terms of the health implications of diets and nutrient scarcity but also in defining the optimal culture conditions in in vitro studies. Just as the composition of the culture medium can alter its activating capacity, temporary changes in nutrient availability can impact its activation status, a fact that must be considered when designing and interpreting in vitro experiments. Here, we assessed, in vitro, the effects of short-term serum deprivation (SD) and repetitive SD cycles on macrophage inflammatory status and response to further understand the potential role of fasting in rheumatic disease management, finding that the effects on macrophage activity can be antagonistic depending on the protocol used.

## 2. Results

### 2.1. SD Modulates Macrophage Phenotype and Its Response to LPS Stimulation

**Figure 1:**
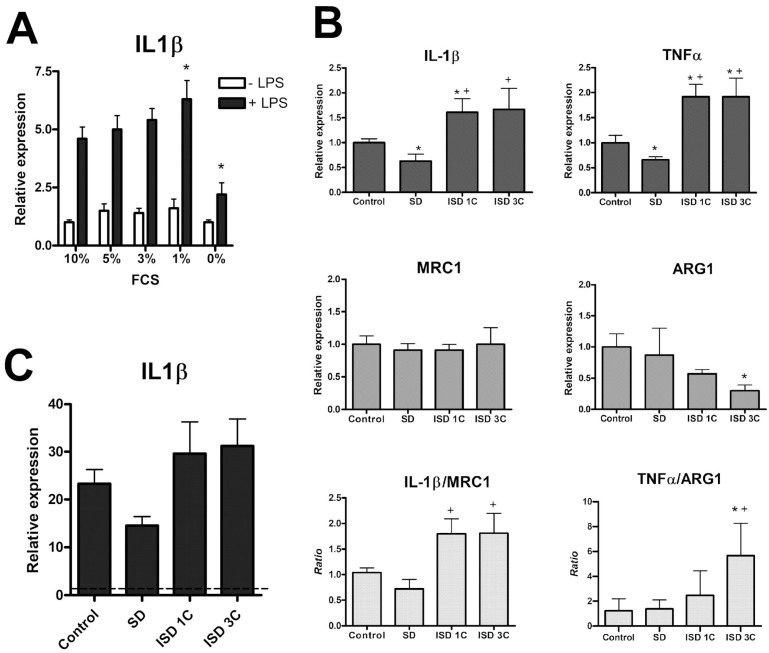
SD modulates the macrophages’ profiles and response to LPS stimulation. (**A**) mRNA expression of IL-1β in macrophages cultured for 3 h at different FCS concentrations and the effect of LPS (100 ng/mL) treatment, as measured by qPCR (*n* = 4). (**B**) Expression of IL-1β (*n* = 3), TNFα (*n* = 4), MRC1 and ARG1 (*n* = 3) in macrophages under different conditions and ratios: IL-1β/MR and TNFα/ARG1 (*n* = 3). (**C**) Gene expression of IL-1β in macrophages stimulated with LPS (100 ng/mL) with different periods of nutrient availability (*n* = 4). Data are expressed as mean ± SEM. * *p* < 0.05 vs. control (10% FCS) group, + *p* < 0.05 vs. SD group. ANOVA with Tukey’s post-test was used to obtain *p*-values. ISD1C, intermittent SD, 1 cycle; ISD3C, intermittent SD, 3 cycles.

In order to assess changes in macrophage pro-inflammatory and anti-inflammatory markers when cultured under different nutrient availability conditions, qPCR analyses were performed. Incubating the macrophages for three hours with different concentrations of FCS (using 10% FCS as the basal level) did not induce substantial changes in the generation of IL-1β in the absence of stimuli (Figure 1A). In contrast, when cells were exposed to LPS, with 0% FCS, the expression of IL-1β in response to LPS was significantly inhibited. Given these findings, subsequent experiments were performed under the complete depletion of FCS.

Changes in macrophage phenotype were studied 24 h after the start of treatments. Macrophages after a 24 h SD (0% of FCS) period showed a significant reduction in IL-1β and TNFα expression; pro-inflammatory markers; and no changes in the anti-inflammatory marker mannose receptor (MRC1) or arginase 1 (ARG1). Consequently, there was a significantly lower IL1B/MRC1 ratio compared with the control group and no changes in the TNFα/ARG1 ratio (Figure 1B). In contrast, 24 h of refeeding after one or three cycles of 1 h SD (intermittent SD, ISD) shifted the macrophage profile toward a pro-inflammatory phenotype, with significantly higher IL-1β and TNFα expression and significantly reduced ARG1 expression only in ISD3C, which resulted in an increased IL1B/MRC1 and TNFα/ARG1 ratio. Anyway, this does not represent a radical change in the phenotype since the intensity of the changes was subtle but consistent.

**Figure 2:**
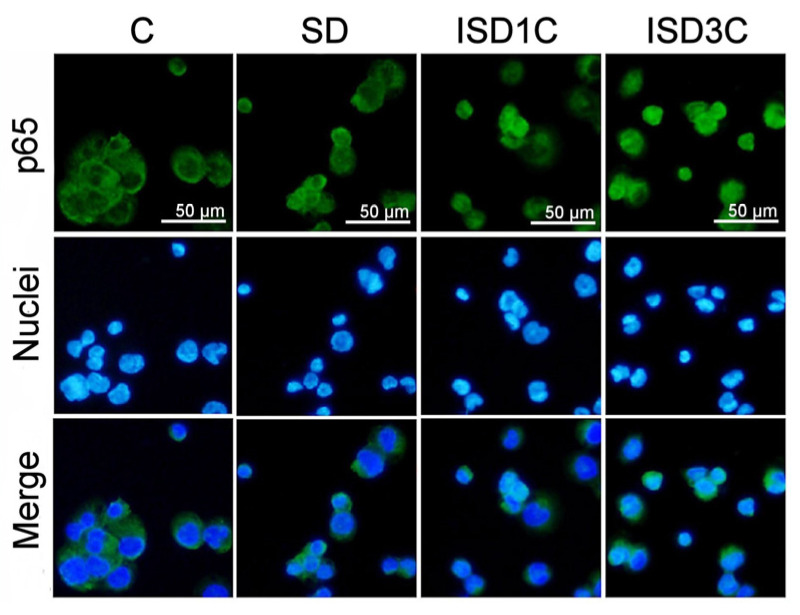
SD effects on NF-κB activation. Immunofluorescence of p65 subunit of NF-κB in macrophages under the FCS conditions described in Figure 1. In control and SD groups, p65 subunit of NFkB remains in cytoplasmatic localization. Nuclear translocation was mainly observed in ISD groups (*n* = 3).

When we assessed the macrophages’ IL-1β response to LPS when undergoing SD for 24 h or 24 h of refeeding after one or three cycles of ISD, the results showed a similar profile to what was observed in the absence of the inflammatory stimulus, that is, a tendency to decrease the expression of IL-1β in SD and an increased response when one or more cycles of ISD was applied, with no significant differences between ISD groups (Figure 1C). Finally, immunostaining p65 was performed in order to assess NF-κB activation by nuclear translocation under the different nutrient availability conditions (Figure 2).

The results show that there were no changes during SD, while ISD cycles generated a slight increase in the nuclear translocation of p65.

### 2.2. SD Affects Macrophage Metabolic Activity

**Figure 3:**
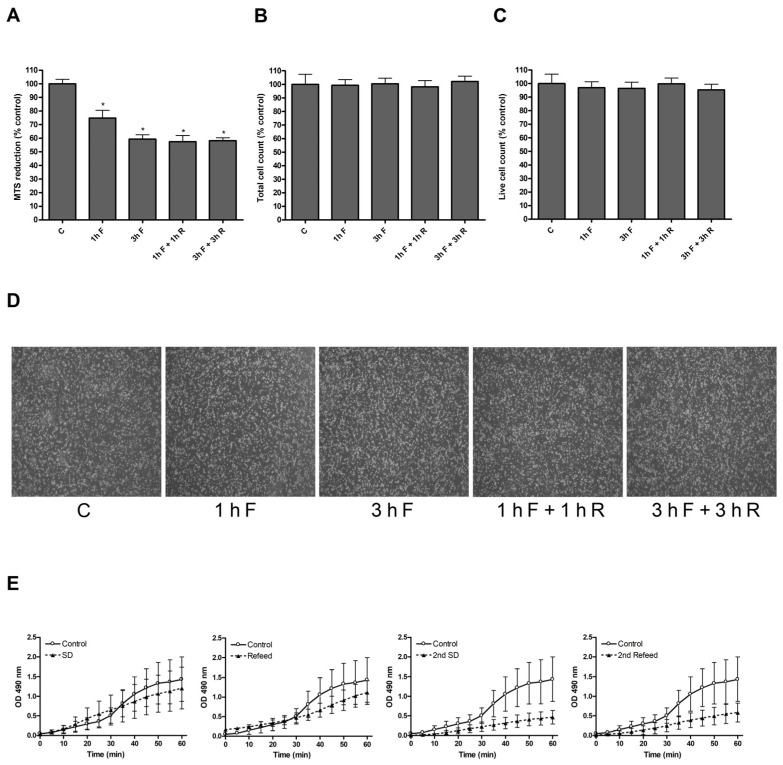
SD affects the metabolic activity of macrophages independently of cell viability. (**A**) MTS assay (*n* = 4); (**B**) total cell number; and (**C**) live cell number of macrophages undergoing different SD and refeeding time periods (*n* = 3). (**D**) Macrophages grown under different SD and refeeding time periods; pictures were taken with a microscope at the end of the treatments. Scale bars represent 200 μm (*n* = 3). (**E**) Kinetics of MTS of macrophages undergoing different SD and refeeding cycles; measurements were performed every 5 min for an hour after each change of media. Data are expressed as mean ± SEM. * *p* < 0.05 vs. control group (*n* = 3). ANOVA with Tukey’s post-test was used to obtain *p*-values. SD, serum deprivation; R, refeeding.

To assess whether the responses of macrophages to different fasting protocols were dependent on cell viability, an MTS reduction assay was performed to measure metabolic activity (Figure 3A).

Compared with the control group, macrophages undergoing both fasting and ISD showed a lowered MTS reduction capability, independently of the length of the treatment. The decrease was observed even at 1 h of fasting, and although it decreased even more with 3 h of fasting or with IF, the difference did not reach significance (Figure 3A), indicating that the changes take place very early in fasting. However, this was not related to a decrease in cell number since, when cell number and viability were evaluated by Evan’s blue exclusion, no changes between groups were detected (Figure 3B,C). Finally, the images obtained under the microscope also show that there were no changes in the number of cells in the different conditions (Figure 3D). These results suggest that SD does not affect macrophage viability, but it does reduce their metabolic activity instead.

To gain a better understanding of the impact of fasting on the metabolic capacity of macrophages, we conducted a study on the kinetics of MTS reduction over shorter time periods (Figure 3E). The results indicate that the rate of MTS reduction caused by macrophages remains stable during the initial hour of fasting, and replenishing fetal serum does not affect this reduction capacity. However, when the SD period is repeated, a decrease in the initial reducing capacity can be observed, and this decline is not reversed even after restoring fetal serum levels.

### 2.3. MTS Reduction and Macrophage Profile Correlate to Metabolic Changes

**Figure 4:**
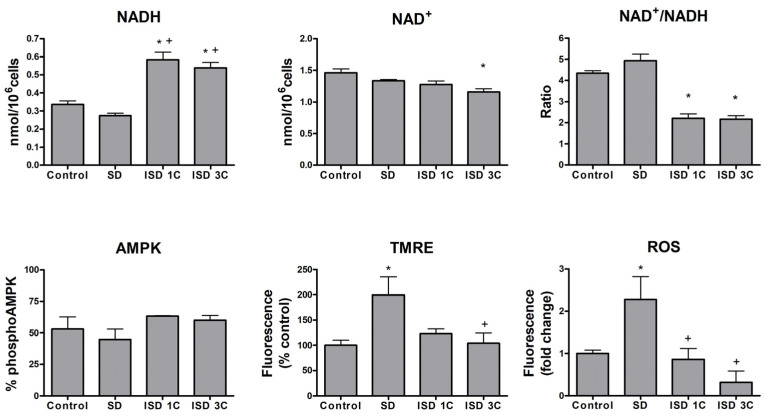
SD and ISD alter cell metabolism caused by regulating mitochondrial dynamics. NADH and NAD^+^ concentrations and NAD^+^/NADH ratio (*n* = 2), phosphorylated AMPK (*n* = 2), TMRE (*n* = 3) and ROS (*n* = 2) under the different SD protocols. Data are expressed as mean ± SEM. * *p* < 0.05 vs. control group; + *p* < 0.05 vs. fasting group. ISD1C, intermittent SD, 1 cycle; ISD3C, intermittent SD, 3 cycles; NAD, nicotinamide adenine dinucleotide; AMPK, 5′ adenosine monophosphate-activated protein kinase; TMRE, tetramethylrhodamine, ethyl ester; ROS, reactive oxygen species.

To further elucidate whether the decreased ability to reduce MTS could be due to metabolic changes in response to nutrient availability, we proceeded to study different mitochondrial activity markers on macrophages undergoing 1 h long SD/refeeding cycles (Figure 4).

A slight decrease in NADH levels was detected during the SD periods, while in the ISD groups, their concentrations increased significantly; these findings suggest that ISD increases the reduction capacity of macrophages. It is interesting to note that this profile is similar to what was detected in the expression of IL1β and TNFα (see Figure 1). Changes in NAD^+^ levels were smaller, and only a significant reduction was detected in the ISD3C group compared with control. Finally, the NAD^+^/NADH ratio increased modestly in the SD group and significantly decreased in ISD. AMPK activation levels were also studied, and again, a similar profile to that of NADH and the pro-inflammatory marker expression was observed. However, in this case, the changes were smaller, and none of them reached statistical significance. Finally, ROS generation and changes in mitochondrial membrane potential (TMRE) were evaluated. In this case, both markers were increased in the SD groups, and these increases reverted in intermittent SD, suggesting that these changes are transitory and strongly associated with limited nutrient availability. These results together confirm that intermittent SD has a different effect on macrophage activity than simple SD, showing higher NAD^+^/NADH levels and reduced oxidation.

## 3. Discussion

Limitation in nutrient intake, and in its most extreme case, fasting has been the subject of great interest for years due to its alleged health benefits [18,19]. Since this is a necessarily time-limited strategy, IF has emerged as a possible alternative to constant fasting regimes that may be difficult to implement and maintain in humans. Recent reports suggest that IF has similar health benefits to normal fasting, including weight loss [19,20], cardiometabolic health [20,21] and improving rheumatic diseases [6,7,8,10,22], among others.

One of the proposed mechanisms by which intermittent fasting may be exerting its beneficial effects on health is through a generic reduction in inflammatory status. However, this has also generated conflicting results, probably due to variabilities in the protocols followed. While some studies have described a reduction in the inflammatory response to intermittent fasting [18,23,24,25,26,27], others have found it not to cause any effects [28,29] or even to be associated with a pro-inflammatory response [30].

Although the effects of fasting have been widely investigated in vivo, there are only a few studies of the in vitro effects of nutrient deprivation [31,32,33,34,35], and not many of them focus on inflammation. Furthermore, there is wide variability in the protocols followed in these studies, not only in the length of the intervention but also in the nutrients present in the culture media mimicking fasting conditions. Our goal was to use a simple model to study, in vitro, the inflammatory response to fasting, so we designed a straightforward intervention based on different levels of nutrient deprivation (as performed through the complete removal of serum in the culture media) for the activity profile of macrophages. This study was performed with THP1 cells differentiated into macrophages. This is a widely used model, especially in the analysis of the inflammatory response. However, keep in mind that they are still cells derived from monocytic leukemia and that they may have a metabolic profile that does not fully reflect that of all types of primary macrophages.

It has previously been shown that macrophage activity can be regulated by diet [36] and that fasting promotes an anti-inflammatory profile [31,32,33,34,35,36,37]. Our initial results are consistent with these studies, as the depletion of serum levels in the medium results in the impaired adoption of the M1 phenotype, evidenced by decreased TNFα expression and IL1β expression, both under basal conditions and in response to LPS (Figure 1A). This decrease could hardly be attributed simply to a limitation in the ability to generate the additional energy necessary to start the cellular machinery associated with the activation of macrophages since a high concentration of glucose was maintained in the media.

On the other hand, serum replenishment after one short SD cycle or three SD/refeeding cycles, which we used as protocols to simulate intermittent fasting (IF), resulted in a shift in the macrophage profile toward a more pro-inflammatory profile. This change is related to IL1β and TNFα expression, while MRC1 remains unmodified, and ARG1 only decreases with ISD (see Figure 1B). These results support studies that suggest that, unlike sustained fasting, an IF regime promotes an inflammatory state [30]. However, MRC1 does not show such a clear profile (see Figure 1B). All of this suggests that changes in nutrient availability may be reflected in the degree of macrophage activation but that the mechanism is more subtle than a simple drift toward a marked pro- or anti-inflammatory state.

Of course, the effects on a single cell type do not reflect the complexity of the changes that can take place throughout the organism, and the effects of fasting vs. IF on inflammatory responses should be further investigated in vivo. It may also be relevant that we evaluated the effects of withdrawing and replenishing nutrients right after a period of refeeding, whereas most studies evaluate their effects at the end of a fasting cycle. Finally, it should be noted that when we studied the response of macrophages to an inflammatory stimulus, such as LPS, during nutrient deficiencies, we confirmed that SD reduces the inflammatory response, whereas one or three cycles of ISD restored the response of macrophages to LPS (see Figure 1C), reinforcing the idea that fasting shifts the profile of macrophages to M2, while the renewed availability of nutrients does so to M1.

The results obtained when we evaluated the activation of NFκB, one of the main transcription factors associated with the inflammatory response, were similar. During fasting, no changes in the cellular localization of the p65 subunit of NFκB were detected (see Figure 2), but after one to three cycles of IF, a certain degree of nuclear translocation was observed, denoting an activation of NFκB that coincides with the observed increase in IL1β and TNFα expression.

Another change we observed after ISD cycles in macrophages is the change in the reducing capacity of these cells. We initially wanted to measure changes in cell viability using the MTS reduction assay, but we quickly realized that the detected decrease in MTS tetrazolium compound reduction—which deepened with each SD cycle and did not recover after longer refeeding periods (see Figure 3A)—did not correspond to what was observed under the microscope, where no decrease in the total number of cells or the percentage of viability measured with trypan blue exclusion could be seen (see Figure 3B,C). It is important to keep in mind that determining cell viability with the MTS reduction method requires that the reducing capacity of the cells does not change as a result of the treatment. Consequently, we concluded that the drop in the levels of reduced MTS after SD/refeeding was not due to a decrease in cell viability but to changes in the ability of macrophages to reduce this molecule. The subsequent MTS kinetic analysis allowed us to assess the changes through shorter periods of time, which showed fast albeit consistent variations in both the SD and ISD groups (see Figure 3D). A single cycle of SD and refeeding has a moderate effect on the reducing capacity, but a second cycle decreases this capacity markedly and is not recovered in the subsequent refeeding. The changes actually begin after 30 min of SD, so this effect must be taken into account when manipulating the macrophage cultures that involve the removal of serum from the culture medium.

The MTS reduction assay depends on cellular NAD(P)H-dependent oxidoreductases, and therefore, the results are affected not only by cell number but also by changes in mitochondrial content and metabolism [38,39]. Reduced MTS capacities are associated with many factors, including reduced NAD(P)H and reduced dehydrogenase enzyme activity [40]. Indeed, our results show a significant reduction in the NAD+/NADH ratio in both IF groups, which could correlate to the MTS results (see Figure 4). These changes are due to an accumulation of NADH while NAD+ levels remained unchanged. These changes are useful for indirectly assessing cellular energy capacity, and our results suggest that both SD and IF induce changes in mitochondrial activity.

Along with a tendency to reduce NADH levels during fasting, a hyperpolarization of the mitochondrial membrane potential was observed. This has been associated with increased fatty acid oxidation (FAO) and could be a mechanism to meet the high energy demands in response to fasting [41]. This metabolic shift supports anti-inflammatory responses, which, interestingly, coincides with what was observed in the expression of IL1β and TNFα during SD (see Figure 1B). Moreover, an increase in ROS was observed during fasting. Macrophages can certainly generate ROS in many situations and in response to different stimuli, but the marked parallelism observed in our experimental conditions between membrane depolarization and ROS generation suggests that these are two aspects of the same mechanism. Preventing mitochondrial depolarization in the presence of high levels of ROS could be a defense mechanism of the cell used to avoid apoptosis in a situation of stress due to a lack of nutrients. Interestingly, mitochondrial membrane potential and ROS were closer to basal levels after the refeeding cycles, which may reflect a return to physiological conditions and the resolution of the insult. After refeeding, the NADH levels increased, which could indicate enhanced glycolysis in response to the nutrient replenishment. This has been associated with a pro-inflammatory profile [42] and relates to what we observed with the levels of IL1β and TNFα. However, an accumulation of NADH in the ISD groups was also observed, and the MTS reduction capacities were not recovered, which indicates that the macrophages are not fully recovered after ISD, and a certain level of mitochondrial dysfunction persists.

We also saw a similar profile to the changes in NADH, although the changes were not significant in the levels of AMPK activation. The limited changes detected in the phosphorylated AMPK levels were surprising, as this pathway is thought to be a nutrient and energy sensor [43], and other studies have seen changes in AMPK activation with both fasting and IF [44,45,46,47]. Moreover, the activated form of AMPK is also thought to promote macrophage M2 polarization [48,49] and to act as a metabolic modulator fomenting FAO in response to nutrient changes [50]. This effect could be achieved here via alternative pathways. Additional studies will be needed to better understand these results.

Taken together, our results indicate that short-term serum deprivation and intermittent cycles of serum deprivation can modulate macrophage phenotypes and their response to inflammatory stimuli in different, and even opposite, ways. These modulatory effects are associated with rapid changes in metabolism and have long-lasting effects, even after nutrient replenishment. These results also emphasize the importance of considering changes in the composition of the culture medium as a possible confounder when performing in vitro experiments since it could alter the inflammatory profile of macrophages. Furthermore, our results indicate that while SD may elicit an anti-inflammatory response in macrophages, refeeding may have the opposite effect, which could help to explain the conflicting results of studies on the health benefits of different fasting/intermittent fasting protocols. This is also important when designing dietary interventions as adjuvants in the control of inflammatory diseases.

## 4. Materials and Methods

### 4.1. Cell Line

Human monocytic THP-1 cells were cultured in a suspension in RPMI-1640 medium supplemented with 10% fetal calf serum (FCS), 100 U/mL penicillin, 100 µg/mL streptomycin and 2.5 μg/mL Amphotericin B. Cells were grown in a humidified incubator, 95% air, at 37 °C under 5% CO_2_. For differentiation into macrophages, cells were treated with 100 nM phorbol 12-myristate 13-acetate (PMA) (Merck, Rahway, NJ, USA) and incubated for 24 h. After washing with Phosphate-Buffered Saline (PBS), the PMA-containing media were replaced with fresh high-glucose Dulbecco’s Modified Eagle’s Medium (DMEM) supplemented with 10% FCS, 100 U/mL penicillin and 100 µg/mL streptomycin, and the cells were incubated for 24 h before starting the treatments.

### 4.2. Cell Treatments

Cell treatments were performed by plating 300,000 cells/well (12-well plate); 200,000 cells/well (24-well plate); 40,000 cells/well (96-well plate); and 50,000 cells/well (8-well culture slide). After differentiation, cells were cultured with supplemented DMEM media 24 h pre-treatment.

Treatments consisted of depriving cells of FCS (0%) for different periods of time (1 h or 3 h) with the subsequent reestablishment of FCS-supplemented DMEM (10%) for varying incubation periods (1 h or 3 h). The repeated SD cycles consisted of interspersed 1 h long incubations with or without FCS. A washing step with PBS was performed in between media replacements. In some experiments, the effect of lipopolysaccharide (LPS) (100 ng/mL) was also tested.

### 4.3. Cell Counting

Cell number and viability were determined by trypan blue dye exclusion. Duplicate wells of cells for each treatment were counted under a microscope with a Thoma cell chamber. For the staining process, cell suspensions were diluted 1:2 with 0.4% trypan blue dye.

### 4.4. MTS Assay

The CellTiter 96 AQueous One Solution Cell Proliferation Assay (MTS) (Promega, Madison, WI, USA) was used according to the manufacturer’s instructions. Then, 30 min before ending the treatments, 20 µL of reagent was added to each well containing 100 µL of medium. At the end of the treatments, absorbance at 490 nm was measured using a 96-well plate reader. For the kinetics of MTS reduction, the MTS reagent was added 1 h before the end of the treatments, and absorbance at 490 nm was measured every 5 min with a 96-well plate reader at 37 °C.

### 4.5. RT-PCR and qPCR

Total RNA was obtained by phenol–chloroform extraction and ethanol precipitation using TRizol^®^ reagent (Invitrogen, Carlsbad, CA, USA). Isolated RNA was diluted in RNAse-free water and stored at −80 °C. RNA concentration was quantified using a Nanodrop ND-1000 (Nanodrop ND-1000 SpectrophoTometer, Thermo Fisher, Waltham, MA, USA) device. A reverse transcription reaction was performed on a 1 μg RNA sample using iScript reagents (Bio-Rad, Hercules, CA, USA) following the manufacturer’s specifications. The mixture was incubated at 25 °C for 5 min; 42 °C for 30 min; and 85 °C for 5 min. Finally, it was diluted in RNAse-free water, obtaining a final concentration of 10 μg/mL. Samples were stored at −80 °C.

Subsequent qPCR amplification was performed using iTaq^®^ SYBR Green Supermix (Bio-Rad, Hercules, CA, USA) and the corresponding primers: IL1β Forward: 5′-GGACAAGCTGAGGAAGATGC-3′ Reverse: 5′-TCGTTATCCCATGTGTCGAA-3′; MRC1 Forward: 5′-GGATGGATGGCTCTGGTG-3′ Reverse: 5′-TCTGGTAGGAAACGCTGGTC-3′; GAPDH Forward: 5′-GATCATGAGCAATGCCTCCT-3′ Reverse: 5′-TGTGGTCATGAGTCGTTCCA-3′; TNFα Forward: 5′-GGCACCACCAACTGGTTATC-3′ Reverse: 5′-AGCCCATGTTGTAGCAAACC-3′; ARG1 Forward: 5′-ACACTCCACTGACAACCACA-3′ Reverse: 5′TCCACGTCTCTCAAGCCAAT-3′. Reactions were performed in duplicate, and threshold cycle values were normalized to GAPDH gene expression. The specificity of the products was determined by melting curve analysis. The ratio of the relative expression of the target genes to GAPDH was calculated by using the ΔC(t) formula.

### 4.6. Immunofluorescence

To monitor p65 translocation, cells were incubated on 8-well culture slides, and after treatments, cells were fixed with 3.5% formaldehyde for 5 min, permeated with 0.1% triton X-100 and blocked with serum. The staining was performed by incubating with NFκB p65 antibody (C-20) (Santa Cruz Biotechnology, Santa Cruz, CA, USA) and secondary antibody goat anti-rabbit AlexaFluor-488 (A11008, Thermofisher) previous to mounting with aqueous medium. Nuclear or cytoplasmic localization was examined by fluorescence microscopy.

### 4.7. NAD^+^ and NADH Quantitation

NAD^+^ and NADH concentrations were quantified with a commercially available kit (no. MAK037, Sigma, St. Louis, MO, USA). The protocol was followed according to the manufacturer’s instructions, and absorbance at 450 nm was measured using a 96-well plate reader.

### 4.8. Phospho-AMPK Alpha 1 and Total AMPK Alpha 1

A phospho-AMPK alpha 1 (S487) and total AMPK alpha 1 ELISA kit (no. ab279734, Abcam, Cambridge, UK) was used to measure human phospho-AMPK alpha 1 and total AMPK alpha 1 from cell lysates after treatments. Instructions were followed according to the manufacturer’s instructions, and absorbance was measured at 450 nm with a plate reader.

### 4.9. Reactive Oxygen Species

Reactive oxygen species (ROS) activity was measured using the DCFDA/H2DCFDA—Cellular ROS Assay Kit (no. ab113851, Abcam, Cambridge, UK) according to the manufacturer’s protocol. Measures were obtained using a fluorescence microplate reader at λEx/λEm = 485/535 nm.

### 4.10. Mitochondrial Membrane Potential

A TMRE Mitochondrial Membrane Potential Assay (no. 786-1313, G-Biosciences, St. Louis, MO, USA) was carried out according to the manufacturer’s instructions in order to quantify changes in cell mitochondrial membrane potential after treatments using a fluorescence microplate reader at λEx/λEm = 549/575 nm.

### 4.11. Statistical Analysis

The GraphPad Prism software v. 4.02 (GraphPad Inc, San Diego, CA, USA) was used for all statistical analyses. Data are presented as mean ± SEM. The comparison among groups was performed by using a two-tailed Student’s *t*-test for the comparison of two groups and a one-way analysis of variance (ANOVA) followed by Tukey’s post-test when comparing three or more groups. Statistical significance was considered when *p* < 0.05.

## Data Availability

All data generated or analyzed during this study are available from the corresponding author upon reasonable request. All the materials are included in the Section 4.
